# An Enrichment of CRISPR and Other Defense-Related Features in Marine Sponge-Associated Microbial Metagenomes

**DOI:** 10.3389/fmicb.2016.01751

**Published:** 2016-11-08

**Authors:** Hannes Horn, Beate M. Slaby, Martin T. Jahn, Kristina Bayer, Lucas Moitinho-Silva, Frank Förster, Usama R. Abdelmohsen, Ute Hentschel

**Affiliations:** ^1^RD3 Marine Microbiology, GEOMAR Helmholtz Centre for Ocean ResearchKiel, Germany; ^2^Department of Botany II, Julius-von-Sachs Institute for Biological Sciences, University of WürzburgWürzburg, Germany; ^3^School of Biological, Earth and Environmental Sciences, Centre for Marine Bio-Innovation and School of Biotechnology and Biomolecular Sciences, University of New South Wales, SydneyNSW, Australia; ^4^Department of Bioinformatics, University of WürzburgWürzburg, Germany; ^5^Center for Computational and Theoretical Biology, University of WürzburgWürzburg, Germany; ^6^Department of Pharmacognosy, Faculty of Pharmacy, Minia UniversityMinia, Egypt; ^7^Christian-Albrechts-Universität zu KielKiel, Germany

**Keywords:** metagenomes, defense, CRISPR, restriction modification, sponge microbiome, seawater

## Abstract

Many marine sponges are populated by dense and taxonomically diverse microbial consortia. We employed a metagenomics approach to unravel the differences in the functional gene repertoire among three Mediterranean sponge species, *Petrosia ficiformis, Sarcotragus foetidus, Aplysina aerophoba* and seawater. Different signatures were observed between sponge and seawater metagenomes with regard to microbial community composition, GC content, and estimated bacterial genome size. Our analysis showed further a pronounced repertoire for defense systems in sponge metagenomes. Specifically, clustered regularly interspaced short palindromic repeats, restriction modification, DNA phosphorothioation and phage growth limitation systems were enriched in sponge metagenomes. These data suggest that defense is an important functional trait for an existence within sponges that requires mechanisms to defend against foreign DNA from microorganisms and viruses. This study contributes to an understanding of the evolutionary arms race between viruses/phages and bacterial genomes and it sheds light on the bacterial defenses that have evolved in the context of the sponge holobiont.

## Introduction

Marine sponges (Porifera) represent the oldest metazoan phylum with a fossil record dating back 580 million years in time (Li et al., 1998). Many sponges host dense and diverse communities of unicellular microorganisms within their tissues (Taylor et al., 2007; Hentschel et al., 2012; Thomas et al., 2016). Based on 16S rRNA gene amplicon sequencing, a recent study observed 1000s of symbiont lineages [operational taxonomic units (OTUs)] within sponges, which are dominated by *Proteobacteria* (mostly *Alpha*- and *Gammaproteobacteria*), *Acidobacteria, Actinobacteria, Chloroflexi, Cyanobacteria, Crenarchaeota*, as well as symbionts of several candidate phyla. Representatives of 41 different phyla were thus far recovered from sponges with representatives of 13 phyla being shared among all sponge hosts (Thomas et al., 2016). Sponges are ecologically important in benthic environments (Bell, 2008). The sponge-associated microorganisms carry out functions related to nutrient cycling including carbon, nitrogen, and possibly sulfur and vitamin metabolism (Taylor et al., 2007; Bayer et al., 2008; Hentschel et al., 2012) as well as to secondary metabolism and chemical defense (Wilson et al., 2014). As sessile filter feeders, sponges are capable of pumping seawater at rates up to 1000s of liters per kilogram of sponge per day (Vogel, 1977; Weisz et al., 2008). Small particles are retained from the incoming seawater and transferred into the mesohyl interior where they are digested by phagocytosis (Bell, 2008; Southwell et al., 2008; Maldonado et al., 2012). Sponges and their microbial consortia (hereafter referred to as the sponge holobiont) are thus continuously exposed to incoming microorganisms, that serve as a food source, but that may also be harmful (Webster, 2007; Wehrl et al., 2007). Despite considerable research effort and several published sponge genomes (Srivastava et al., 2010; Ryu et al., 2016), little is known as to how the sponge holobiont protects itself against potentially harmful microorganisms, whether eukaryotic, prokaryotic, or viral in nature.

One major line of prokaryotic defense is based on the self – non-self-discrimination principle, which recognizes and targets foreign DNA (Makarova et al., 2013). It comprises various systems, among them the clustered regularly interspaced short palindromic repeats (CRISPR). CRISPRs are based on conserved repeats and variable spacer sequences which are incorporated into the host genomes upon encounters from viruses or phages and plasmids and are thus able to memorize the attack (Horvath and Barrangou, 2010). Hence, it is described as the adaptive immune system of prokaryotes (Makarova et al., 2013). Structurally, CRISPRs are associated with *cas* genes, which are essential for their function and which are also used for the CRISPR classification (Makarova et al., 2011). Additional defense systems are the RMS and the DNA phosphothiolation (DND) system (Makarova et al., 2013). The RMS is nearly ubiquitous among bacteria (Vasu and Nagaraja, 2013). RMS can be classified into types I–IV depending on their subunits, recognition sites, cleavage positions, and substrate specificities (Roberts et al., 2003). Both, the RMS and DMD systems, make use of labeling own DNA, either by methylation or by phosphorothioation, and recognize and destroy unmodified non-self DNA (Wang et al., 2007; Vasu and Nagaraja, 2013). The Phage growth limitation (Pgl) system is another line of defense that allows phage burst upon initial infection. In *Streptomyces coelicolor* A(3)2, PgI was shown to target phage Φ31 and its relatives. Here, the DNA of the phage progeny was methylated, which resulted in activation and consequently, in prevention of phage growth through presumed methyl-specific restriction endonuclease activity (Abedon, 2012; Hoskisson et al., 2015). The PglZ protein family is a central element of Pgl, however, the mechanisms of this complex system are poorly understood (Makarova et al., 2013). Another major line of defense is based on dormancy or programmed cell death (Makarova et al., 2013). These can be separated into toxin–antitoxin (T–A) systems and abortive infection (ABI). In the T–A system, the protein toxin kills cells above a certain expression level. The antitoxin component then regulates and/or inactivates toxin expression and prevents killing of the cell. The ABI system is also based on cell death or dormancy and it is also based on two modules (Fineran et al., 2009). The ABI system activates cell death to prevent viral replication and thereby protects the bacterial population.

In the present study we aimed to characterize defense systems of marine sponge-associated microbial consortia. The microbial metagenomes of three Mediterranean sponges (*Petrosia ficiformis, Sarcotragus foetidus, Aplysina aerophoba*) and seawater were compared toward this goal. Besides insights into the microbial community composition and overall GC content, we present defense-related features that consist of the CRISPR system, restriction modification, phage growth inhibition, and genes related to DNA phosphothiolation. The results of the present study are consistent with the concept of “functional convergence” (Fan et al., 2012) that shows similar functional profiles in the microbiomes of different sponge species and that are distinct from those of seawater.

## Materials and Methods

The sponges *P. ficiformis* (sample ID: 1Biotec2_S07) and *S. foetidus* (sample ID: 1Biotec2_S06) were collected on 25 May 2013, by SCUBA diving in Milos, Greece (N36.76759° E24.51422°), at 5–7 m depth. Sponge tissues (5 ml each) were washed with sterile-filtered seawater, passed through a 100 μm Nitex cloth (Hartenstein, Germany) and transported to the laboratory in glycerol solution (15% v/v) at -20°C until further processing. A total of 10 L seawater (sample ID: Biotec_SW) was collected from the vicinity of the sponges. Within 2–3 h after collection, seawater was filtered consecutively through 100 μm Nitex (Hartenstein), 5 μm durapore (Merck-Millipore), and finally through 0.22 μm durapore membrane filters, which were then frozen at -20°C.

Sponge samples of *A. aerophoba* were collected in the Mediterranean Sea from a depth of 5 m (Piran, Slovenia), on 07 May 2013. Upon transport back to the laboratory, samples of pinacoderm and mesohyl were separated using a sterile scalpel blade. One scalpel blade was used per each sample to prevent cross-contamination between samples. Microbial cells were enriched from the different sponge tissues by differential centrifugation (Fieseler et al., 2006). Microbes from *P. ficiformis* and *S. foetidus* samples were prepared using the same protocol. Fractions of sponge-associated prokaryotes (SAPs) were frozen at -80°C in 15% glycerin.

### DNA Extraction and Sequencing

Genomic DNA was extracted from the sponge SAP preparations of *P. ficiformis* and *S. foetidus* and the seawater filters using the FastDNA Spin Kit for Soil (MP Biomedicals, USA). The quantity of metagenomic DNA was determined by spectrophotometry using a NanoDrop 2000c reader (PEQLAB Biotechnologie GmbH, Germany). The quality and size were analyzed by visual inspection on 0.8% agarose gels following electrophoresis.

DNA of *A. aerophoba* was extracted in triplicates for each pinacoderm and mesohyl using the FastDNA SPIN Kit for Soil (MP Biomedicals). In order to maximize DNA yield from bacteria with different cell properties, the cell lysis step varied for the three replicates of each tissue type: (i) bead beating, following the manufacturer’s protocol, (ii) freeze-thaw cycling (three cycles of 20 min at -80°C and 20 min at 42°C), (iii) proteinase K digestion (bacterial pellet re-suspended in 567 μl TE with SDS in a final concentration of 0.5% and proteinase K in 100 ng/ml final concentration) for 1 h at 37°C. After cell lysis, the manufacturer’s protocol was followed for all six samples. Extracted metagenomic DNA from *A. aerophoba* samples was sequenced on an Illumina HiSeq2000 platform (150 bp paired-end reads) and quality filtered at the DOE Joint Genome Institute (Walnut Creek, CA, USA). Seawater, *P. ficiformis* and *S. foetidus* derived DNA was sequenced at GATC Biotech AG (Cologne, Germany) on an Illumina MiSeq Personal Sequencer (250 or 300 bp paired-end reads, respectively).

### Raw Data Processing and Assembly

The raw reads obtained for the samples of *P. ficiformis, S. foetidus*, and seawater were initially analyzed with FastQC 0.11.2^1^ for adapters, overall quality, length and ambiguous bases. In a first step, the reads were trimmed using Trimmomatic 0.31 (PE -phred 33 LEADING:3 ILLUMINACLIP:2:30:10) (Bolger et al., 2014) and then merged using bbmerge^2^. All reads, merged and unmerged, were again subjected to Trimmomatic for further quality trimming and length filtering (SE -phred 33 SLIDINGWINDOW:4:25 MINLEN:150 AVGQUAL:30). The remaining reads were assembled with IDBA-UD 1.1.1 (-mink 10 -maxk 100) (Peng et al., 2012). Contigs with a length ≤1000 nt were discarded. The reads obtained for the *A. aerophoba* dataset were processed via the IMG/ER webserver (Markowitz et al., 2012). Quality filtered reads were normalized using bbnorm and assembled with SPAdes 3.5.0 (-only-assembler, -k 21,33,55,77,99,127, -sc) (Bankevich et al., 2012). Only contigs ≥1000 nt were used for further analysis. To remove eukaryotic contamination, all contigs, that were further analyzed, were subjected to blastn 2.2.28 (e-value 10e-6 -task blastn) (Altschul et al., 1990) and searched against the NCBI nucleotide database (nt, as of September 29, 2015). The blast hits were analyzed with Krona 2.6 (Ondov et al., 2011). All reads of eukaryotic origin were removed. Information about the metagenomics datasets is presented in **Tables 1** and **2**.

**Table 1:** Samples analyzed in this study.

	Seawater	*Petrosia ficiformis*	*Sarcotragus foetidus*	*Aplysina aerophoba*
Sample date	29.05.2013	29.05.2013	29.05.2013	07.05.2013
Location	Mediterranean Sea, Milos, Greece	Mediterranean Sea, Milos, Greece	Mediterranean Sea, Milos, Greece	Mediterranean Sea, Piran, Slovenia
Depth	5–7 m	5–7 m	5–7 m	5 m
Temperature	20°C	20°C	20°C	18°C

**Table 2:** Statistics on the processing of the metagenomics samples from sequencing throughput to analysis.

	Seawater	*Petrosia ficiformis*	*Sarcotragus foetidus*	*Aplysina aerophoba*
Sequencing platform	Illumina MiSeq (2 × 300 bp)	Illumina MiSeq (2 × 250 bp)	Illumina MiSeq (2 × 300 bp)	Illumina HiSeq (2 × 150 bp)
Sequenced reads (#)	40,505,000	41,383,600	32,672,426	945,906,728
Sequenced bp	12,151,500,000	10,345,900,000	9,801,727,800	283,772,018,400
Reads after QC (#)	18,273,997	29,213,518	17,525,606	–
Bp after QC	6,240,860,642	7,655,556,186	4,909,483,386	–
Assembly algorithm	IDBA-UD	IDBA-UD	IDBA-UD	SPAdes
Assembly size (bp)	216,407,276	226,772,563	190,159,175	489,999,481
Contigs > 1000 bp	116,626	82,740	41,164	110,609
N_50_ contigs (bp)	1,853	3,381	9,706	8,958
Largest contig	58,177	342,148	369,775	1,056,271
Average %GC	41	63	63	58
Open reading frames	215,442	221,522	175,356	455,396
ORF with COG annotation	129,900	119,914	103,075	203,692
ORF with Pfam annotation	78,569	55,478	29,253	56,643
ORF with TIGRFAM annotation	28,017	17,214	10,675	17,267
Average genome size (bp)	1,347,075.38	3,034,048.52	3,744,502.76	5,165,191.54
Reads mapped to assembly	13,396,184	15,900,219	22,478,672	537,464,688
Bp mapped to assembly	3,642,606,507	3,539,701,337	5,378,691,619	80,619,703,200
Average coverage	16.83	15.61	28.29	164.53

### Taxonomic Affiliation of Reads and Contigs

The processed reads were submitted to MG-RAST with enabled screening for human contamination and disabled dynamic trimming (Meyer et al., 2008). Contigs obtained from the metagenomic assemblies were assigned to taxonomy using blastx 2.2.28 (e-value 10e-6) and the NCBI non-redundant protein database (nr). All hits were submitted to *blast2lca* (default parameters), a last common ancestor algorithm implemented in MEGAN5 (Huson et al., 2011).

### Comparison of GC Content and Average Genome Sizes

The GC content of all four metagenomes was calculated for all processed and filtered reads, using an in-house perl script. In addition, the average genome size per metagenome was computed with MicrobeCensus 1.0.7 (Nayfach and Pollard, 2015) using the same reads and their average calculated length (**Table 1**).

### Data Normalization

Processed reads were mapped to their respective assembly using bowtie2 2.2.4 (very-sensitive) (Langmead and Salzberg, 2012). The coverage for each position on a contig was calculated with samtools depth 1.2 (Li et al., 2009). With this data, the coverage of each contig was set as the mean coverage over each position. To account for the different sequencing depths, the number of mapped reads and assembly size, the coverage for each contig was divided by the total number of mapped basepairs and multiplied by 10^6^ to obtain copy numbers per megabase (cpm).

### Functional Annotation

All contigs were subjected to Prodigal 2.6.0 (-p meta, -c, -g 11) (Hyatt et al., 2010) to predict open reading frames (ORFs). Clusters of Orthologous Groups (COGs) obtained from the Conserved Domains Database (CDD) (Marchler-Bauer et al., 2015) were annotated using rpsblast 2.2.28 (e-value 10e-6). Protein families (Pfam) and TIGRFAM were assigned with the InterProScan pipeline 5.17 (Jones et al., 2014) based on the best hit (e-value 10e-6).

### Characterization of CRISPR Arrays, Repeats, and Spacers

The presence of CRISPR arrays was analyzed with a multiple tool approach similar as proposed by Gogleva et al. (2014) using CRT, PILER-CR, and CRISPRFinder (Bland et al., 2007; Edgar, 2007; Grissa et al., 2007b). *Cas* genes were identified by subjecting ORFs of CRISPR-containing contigs to TIGRFAM and Pfam databases using InterProScan. Assignment of CRISPR-Cas types was accomplished according to Makarova et al. (2011) using the TIGRFAM and Pfam annotations. Contigs containing CRISPR arrays found with CRT and PILER-CR or included *cas* genes were uploaded to CRISPRfinder and were validated as true hits. Of these, only confirmed CRISPR with at least two spacers were retained. Possible targets of spacers were identified by submitting their sequences to CRISPRtarget using the ACLAME (as of August, 2009), GenBank-Phage, GenBank-Plasmid and RefSeq-Viral databases (all as of September, 2015) (gap open -5, gap extend -2, nucleotide match +1, mismatch -1, e-value 0.1, word size 7) (Biswas et al., 2013). Direct repeat seqences were submitted to CRISPRdb (Grissa et al., 2007a) and blasted against the CRISPRfinder database (e-value 10e-2) and CRISPRmap (Lange et al., 2013) to examine their superclasses by sequence and structure and to determine if they were reported before. The origin of the CRISPR arrays was determined through their respective contigs as described in Section “Taxonomic Affiliation of Reads and Contigs.”

### Analysis of Restriction Modification Systems

Reference protein sequences of type I [restriction endonucleases (REase), methyltransferases (MTase), and specificity domains], type II (REases ant MTases), and type III (REases and MTases) RMSs) were downloaded from REBASE (as of October 15, 2015) (Roberts et al., 2015). For each type of REases, MTases and specificity domains, a blast database was built. Predicted ORFs from all metagenomes were queried against the databases using blastp 2.2.28 (e-value 10e-6) and only hits with a coverage ≥70% were kept. A RMS was considered as being complete, if its restriction endonuclease and methyltransferase were at least four genes apart from each other (Oliveira et al., 2014). Finally, overlapping regions of REases and MTases (and specificity domains for type I) of the same type within four genes were combined to one cluster to avoid double counts.

### Deposition of Sequence Data

The sequencing projects were completed in 2013 and sequencing data was deposited in the Sequence Read Archive (SRA), metagenome assemblies as a Whole Metagenome Shotgun (WGS) projects in GenBank under the BioProject PRJNA318959 and the BioSample IDs SAMN04870510, SAMN04870527, SAMN04870528 and SAMN05860141 for *P. ficiformis* (SRA: SRP074318, WGS: LXNJ00000000), *S. foetidus* (SRA: SRP074318, WGS: LXNI00000000), seawater (SRA: SRP074318, WGS: LXNH00000000), and *A. aerophoba* (WGS: MKWU00000000). Raw sequencing data of *A. aerophoba* is available under the GOLD Study ID Gs0099546 ^3^ with the GOLD Project IDs Gp005580–Gp005585 which can be downloaded via the JGI Genome Portal.

## Results

### Sample Description

Three samples from the sponges *P. ficiformis, S. foetidus*, and *A. aerophoba* as well as one seawater sample from the Mediterranean Sea were investigated in this study for functional differences of their associated microbiomes (**Table 1**). Using Illumina MiSeq and HiSeq platforms, more than 1,064,000,000 high-quality sequences (∼310 Gbp) were generated. The metagenomes had assembly sizes ranging from 190 to 489 Mbp. The predicted ORFs ranged from 175,356 in *S. foetidus* up to 455,396 in *A. aerophoba.* A total of 44.73–60.29% could be annotated via COGs (**Table 2**). In order to compare the generated data, the metagenomes were normalized based on their coverage which ranged from 15.61- to 164.53-fold.

### Genomic Composition

**FIGURE 1:**
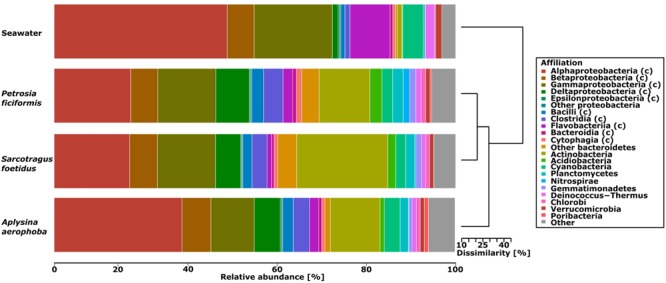
**Barplot showing the relative genomic diversity and associated hierarchical clustering of the metagenomic samples using the Bray–Curtis dissimilarity and complete linkage.** The relative abundance is scaled on the x-axis. Taxonomic assignments are based on MG-RAST annotated phylum and class level (indicated by (c)). For the groups contributing ≥5% of relative abundance (*Proteobacteria, Bacteroidetes, Firmicutes*) class level assignments are given. The group ‘other’ comprises eukaryotes, viruses, archaea, fungi, bacteria at ≤1% abundance and unclassified sequences.

Based on phylogenetic affiliations using the lowest common ancestor algorithm (LCA) in MG-RAST, over 95% of the reads of all four metagenome samples were assigned to 38 bacterial phyla. Proportions of archaea, eukaryotes, viruses and unclassified sequences were consistently low (each ≤ 2.72%) in all metagenomes and were thus not further analyzed (Supplementary Figure S1). The genomic composition of the metagenomes was then analyzed on the phylum and class level. As indicated by Bray–Curtis dissimilarity, the metagenomes of *P. ficiformis and S. foetidus* were closest to each other (11.18% dissimilarity). Both showed dissimilarities of 17.01 and 19.46% to *A. aerophoba*. The seawater sample displayed dissimilarities of 41.04, 43.55, and 31.26% to *P. ficiformis, S. foetidus*, and *A. aerophoba*, respectively (**Figure 1**).

A limited number of sequences (0.15–0.21%) was not assigned to any known bacterial taxa. The *Proteobacteria, Firmicutes*, and *Bacteroidetes* were the most abundant phyla in all metagenomes. The *Actinobacteria* (12.3–22.63% vs. 1.18%) and the *Deltaproteobacteria* (6.28–7.24% vs. 1.38%) were more abundant in the sponge samples than in seawater. In contrast, the *Alphaproteobacteria* were less abundant in sponges than in seawater (18.80–31.81% vs. 43.11%), and so were the *Flavobacteria* (1.12–2.43% vs. 9.99%) and the *Cyanobacteria* (2.49–3.96% vs. 4.98%). Only minor differences between the sponge and seawater samples were found for the *Gammaproteobacteria* (10.77–14.48% vs. 19.51%), the *Clostridia* (3.75–4.86% vs. 1.25%) and other unclassified *Bacteroidetes* (1.38–4.74% vs. 0.43%) according to a principal component analysis (Supplementary Figure S3). Overall, the sponge metagenomes were taxonomically distinct from the seawater metagenome based on taxonomic read assignment using MG-RAST, Bray–Curtis dissimilarity, and principal component analysis.

### GC Footprint

**FIGURE 2:**
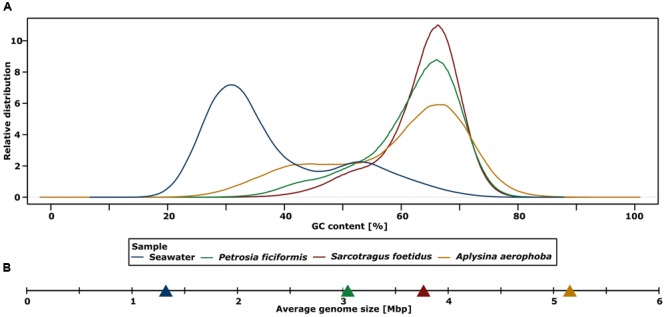
**(A)** Plot of metagenomics samples showing the relative distribution of the GC content of filtered reads. **(B)** Calculated average genome sizes for bacteria of each metagenomic sample.

Higher average GC contents were detected for the assembled metagenomes of sponges (58–63%) than for seawater (41%) (**Figure 2A**; **Table 2**). The highest GC content was detected for the metagenome sample of *S. foetidus*, followed by *P. ficiformis, A. aerophoba*, and seawater. Interestingly, a second smaller seawater peak around 50–55% overlapped with the lower GC tail ends of the sponge metagenomes. To test whether there is a correlation between high GC content and genome size, we calculated the average genome sizes for a bacterial cell within each metagenome. The calculated average genome sizes in the sponge sample were considerably higher than those of the seawater sample (**Figure 2B**; **Table 2**).

### General Functional Properties

**FIGURE 3:**
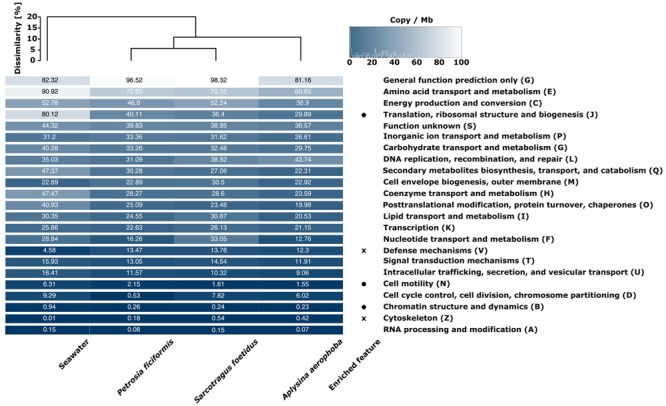
**Heatmap of COG functional categories for the four analyzed metagenomes.** The color scale ranges from 0 (black) to 100 (white) and indicates copies per megabase metagenome (cpm). Functional dissimilarities (Bray–Curtis) are indicated by the dendrogram on top. The term “enriched feature” relates to COG classes which are on average at least 1.5-fold higher in seawater (circle) or in sponges (cross) over all sponge samples. COG classes are ordered from high to low copy numbers.

Functional analysis was based on COG assignments. All hits were normalized to copy number per megabase based on their contig coverage. We identified 103,075–203,692 COG hits for the metagenomes which corresponds to 44.73–60.29% of annotated ORFs (**Table 2**). This number includes however the general function (G) and unknown function (S) categories (10.9–21.2 and 5.9–6.9%, respectively). The functional profiles of the sponge samples were more similar to each other than to seawater, as reflected by a Bray–Curtis dissimilarity of 10% between sponge and seawater metagenomes. Overall, many genes relate to the COG categories general function (G) or unknown function (S), and most of the COG categories were neither enriched for sponges nor the seawater metagenomes (“enriched” is defined as >1.5-fold more copies per megabase) (**Figure 3**). The category nucleotide transport and metabolism (F) was exceptionally high in *S. foetidus* (33.05 cpm), whereas the category of cell cycle control, cell division, chromosome partitioning (D) was exceptionally low in *P. ficiformis* (0.53 cpm) when compared to the other sponge metagenomes. Only few differences were identified between seawater and sponge metagenomes based on COG level assignments. The sponge metagenomes showed a higher number of genes assigned to functions related to defense mechanisms (V) and the cytoskeleton (Z), suggesting that these are important functional traits for sponge symbionts. On the other hand, fewer reads were assigned to translation, ribosomal structure and biogenesis (O), cell motility (N), and chromatin structure and dynamics (B) in the sponge sample, marking them as relevant functional features for free living bacteria.

### Defense Mechanisms

#### COG and Pfam-Annotated Defense Mechanisms

**FIGURE 4:**
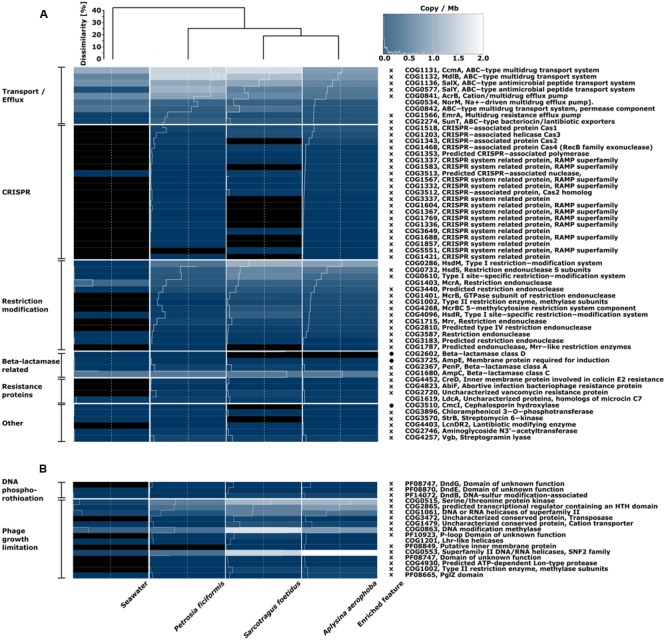
**Heatmap of defense mechanisms in **(A)** COG functional categories and **(B)** additional searches for the phage growth limitation and DNA phosphorothiotation in the COG and Pfam databases.** The color scale ranges from 0 (black) to 2 (white) and indicates copies per megabase metagenome. Bray–Curtis dissimilarity is indicated by the dendrogram on top. Enriched feature relates to COG classes which are on average >1.5-fold higher in seawater (circle) or in sponges (cross) over all sponge samples. Similar COG annotations are labeled on the left side of the heatmap and ordered from high to low copy numbers.

With respect to defense mechanisms, the sponge datasets were more similar to each other than to seawater according to Bray–Curtis dissimilarity measure (**Figures 4A,B**). All comparisons are based on copies per megabase (cpm). Features were defined as “enriched” when being >1.5-fold abundant in either the sponge or seawater metagenome. Transport and eﬄux systems for drugs were found in all samples, and with the exception of a Na^+^-eﬄux pump and ABC-type multidrug transporter, all related functions were enriched in the sponge samples over seawater. Furthermore, all annotations associated to CRISPR were enriched in the sponge microbiomes, as all (with the exception of one CRISPR-nuclease (COG3513) were absent from seawater. 11 features related to CRISPR in *S. foetidus* and one in *P. ficiformis* were missing from the sponge metagenomes, which were mostly related to the receptor activity-modifying proteins (RAMP) superfamily. Interestingly, the cas2-gene (COG1343) in *S. foetidus* may be substituted by a cas2-homolog (COG3512), which showed highest cpm within this metagenome. With respect to RMSs, all genes except one encoding for one endonuclease (COG1403) were enriched in the sponge datasets. However, one endonuclease (COG1787) copy was absent in *S. foetidus* and six were absent from seawater. The overall cpm’s within the RMS were higher in sponge metagenomes than in the corresponding seawater metagenome. Classes A and C beta-lactamases (COG2367, COG1680) were further enriched in sponge metagenomes. On the other hand, seawater was enriched for the beta-lactamase class D (COG2602) and an inductive membrane protein (COG3725). A couple of genes related to resistance against colicin COG4452), a growth inhibiting toxin, bacteriophages (COG4823) and the antibiotic vancomycin (COG2720) were enriched in sponge metagenomes, whereas a cephalosporin hydroxylase (COG3510) was more abundant in the seawater metagenome (**Figure 4A**).

The overall gene copy numbers for DNA phosphorothioation (DND) and phage growth limitation (Pgl) were higher in the sponge than in the seawater metagenome (**Figure 4B**). The DndG (PF08747) was absent from seawater. With respect to the Pgl system, two core genes (COG1002, COG4930) and three additional genes (PF08849, PF10923, COG3472) were missing from seawater. The overall Pgl gene copy number in sponges was ∼50% higher in *A. aerophoba* and *S. foetidus* than in *P. ficiformis* (**Figure 4B**).

#### Clustered Regularly Interspaced Short Palindromic Repeats

**FIGURE 5:**
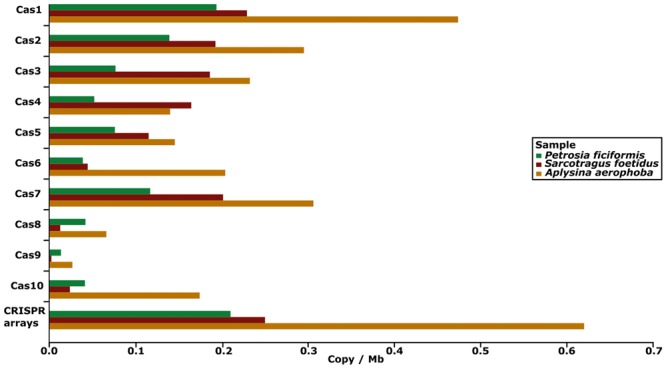
**Barplot showing the abundance of CRISPR arrays and *cas* genes in the four metagenomes.** The x-axis shows their abundance in copy number per megabase.

We analyzed CRISPR arrays and related components, i.e., direct repeat sequences separated by spacers and adjacent *cas* genes in the four metagenomes. The highest numbers of CRISPR array containing contigs were found by searching for *cas* genes. *A. aerophoba* showed the highest abundance of validated arrays, whereas none was identified in the seawater metagenome. The final number of identified CRISPR arrays was 77 (0.21 cpm), 47 (0.25 cpm), 283 (0.62 cpm), and 0 (0 cpm) for the metagenomes of *P. ficiformis, S. foetidus, A. aerophoba* and seawater, respectively (**Table 3**; **Figure 5**). On the domain level, taxonomy was assigned to 53 of 77 (68.83%) arrays in *P. ficiformis*, 40 of 47 (85.11%) arrays in *S. foetidus* and 240 of 283 (84.81%) arrays in *A. aerophoba*. Noteworthy, despite the differences in their geographic location and total number of identified arrays, a large overlap of taxonomic groups was found. The overall distribution of taxa containing CRISPR-contigs was similar in the three sponge datasets, with *Proteobacteria* as the most prevalent phylum followed by *Actinobacteria* and *Chloroflexi* in the sponges *P. ficiformis* and *S. foetidus* and *Firmicutes* in *A. aerophoba* (**Table 3**).

**Table 3:** Raw counts of identified CRISPR arrays and their taxonomic assignments, *cas*-genes, spacers and direct repeats.

	Seawater	*Petrosia ficiformis*	*Sarcotragus foetidus*	*Aplysina aerophoba*
Pilerr-cr	5	90	76	384
CRT	9	108	83	529
Contigs with found Cas-genes	2	124	101	263
CRISPRFinder	0	77	47	290
CRISPR per megabase	0	0.21	0.25	0.62
CRISPR with assigned taxonomy	0	53	40	240
*Proteobacteria*	0	36	22	169
*Actinobacteria*	0	9	6	33
*Chloroflexi*	0	3	4	6
*Cyanobacteria*	0	1	1	6
*Firmicutes*	0	1	2	14
*Acidobacteria*	0	2	1	4
*Verrucomicrobia*	0	1	2	0
*Bacteroidetes*	0	0	1	7
*Deinococcus–Thermus*	0	0	1	3
CRISPR assigned to CAS-genes	0	26	20	144
Type I (A–F)	0	11	10	73
Type II (A–C)	0	1	2	10
Type III (A–B)	0	1	0	6
Type unknown	0	13	8	55
Largest array (# spacer)	0	112	67	169
Total number of spacer	0	1,366	723	9,669
Unique spacer	0	1,349	714	9,547
Spacer with found target	0	278	152	1,642
Phage	0	55	42	255
Virus	0	19	10	146
Plasmid	0	204	100	1,241
Target unknown	0	1,088	581	8,027
Total number of repeats	0	77	47	290
Number of unique repeats	0	67	40	218
Repeats with hits to CRISPRdb	0	55	25	144
CRISPRmap superclass A/B/C/D/E/F	0 – 0/0/0/0/0/0	47 – 1/7/19/2/6/2	21 – 0/6/8/0/5/2	88 – 3/30/36/0/23/7

Different CRISPR-Cas types were categorized by their associated *cas* genes. In the metagenomic datasets, at least 26 (35.06%), 20 (42.55%), and 144 (46.78%) of the CRISPR arrays were adjacent to *cas* genes for *P. ficiformis, S. foetidus* and *A. aerophoba*, which indicates that these arrays might be complete (**Table 3**). The *cas* genes of all known CRISPR-Cas types were identified. CRISPR-Cas type I was the most abundant, with the subtypes I-E and I-C as the most prevalent for all metagenomes, followed by types II and III. Around 50% of *cas* genes could not be annotated in more detail (type unknown, **Table 3**). According to the number of *cas* genes per megabase, *cas1, cas2* were most abundant, followed by *cas3, cas4* and c*as7* in all three sponge metagenomes. Smallest proportions were detected for *cas8* and *cas9* (**Figure 5**). Even though CRISPR arrays were not identified in the seawater metagenome, two *cas6*-genes were detected.

Spacers are the functional part of the CRISPR defense that recognizes foreign DNA fragments. In the *P. ficiformis* metagenome, a set of 1,366 spacers was detected, of which 1,349 were unique (**Table 3**). The largest CRISPR array in *P. ficiformis* contained 112 spacers. For the *S. foetidus* metagenome, a total of 723 spacers was identified, of which 714 were unique. Here, the longest array contained 67 spacers. Thirdly, in the *A. aerophoba* metagenome, a total of 9,669 spacer sequences were detected with 125 of these occurring more than once and with 9,547 being unique. The longest array found in *A. aerophoba* comprised 169 spacers. None of the spacer sequences were shared between the metagenomic samples suggesting that the three sponge microbiomes have their own distinct CRISPR systems.

**FIGURE 6:**
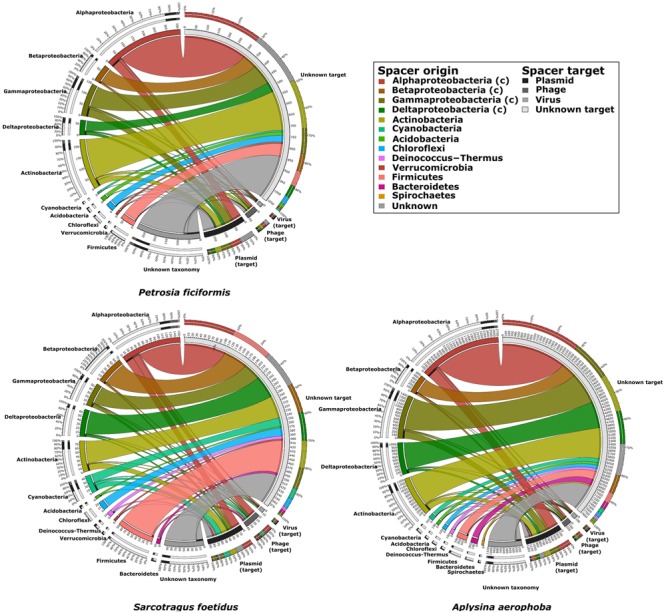
**Plots showing the origin (left side of circles) and targets (right side of circles) of spacer sequences for the three sponge datasets.** The two outermost rings indicate the percentage of target found for each spacer and vice versa. The inner ring indicates the number of spacers connected to the origin and target, respectively.

With respect to potential targets of the spacers, the number of hits decreased from unknown targets, to plasmids, phages and to viruses in all samples (**Table 3**). Combining these results with the spacer taxonomy, most spacers originated in *Alphaproteobacteria* and *Actinobacteria*. All taxonomic groups of spacers had hits in the four target groups, with the largest amount of hits found for unknown targets. The three sponge metagenomes were shaped similarly with respect to spacer origins and targets (**Figure 6**). The proportion of spacer sequences originating from *Betaproteobacteria* was highest in *S. foetidus*, whereas *Gamma*- and *Deltaproteobacteria* were highest in *A. aerophoba*. *S. foetidus* showed more spacers originating from *Firmicutes* than the other sponge samples. Spacers from *Spirochaetes* were only found in the *A. aerophoba* metagenome. Overall, the distribution of spacers with assigned taxonomy followed the genomic composition with correlation coefficients of 0.64, 0.59, and 0.88 (all *p*-values ≤ 0.05) for *P. ficiformis, S. foetidus*, and *A. aerophoba*, respectively (**Figures 1** and **6**). All spacers and direct repeat sequences are compiled in Supplementary Figures S2 and S3.

The number of unique direct repeats was 67, 40, and 218 for *P. ficiformis, S. foetidus*, and *A. aerophoba*, respectively (**Table 3**). In the datasets *A. aerophoba* and *S. foetidus*, three of the direct repeat sequences were shared, of which nine between *P. ficiformis* and *A. aerophoba*, suggesting a horizontal transfer of either CRISPR arrays or bacteria. An amount of 55 (81.09%), 25 (62.5%), and 144 (66.06%) of *P. ficiformis, S. foetidus*, and *A. aerophoba* derived repeats were assigned to known repeat sequences using CRISPRdb. With respect to the classification using CRISPRmap, 47 (70.15%), 21 (52.5%), and 88 (41.12%) direct repeats for *P. ficiformis, S. foetidus*, and *A. aerophoba* were assigned to known superclasses, with the most abundant classes C, E, and B (**Table 3**). Notably, the superclasses were ordered decreasing in their conservation (Lange et al., 2013), showing a mixture of repeats with a roughly corresponding structure (superclasses B and C) and little sequence conservation (superclass E). Overall, 81.09 and 70.15% of all direct repeat sequences could be classified using CRISPRdb and CRISPRmap, respectively.

#### Restriction Modification Systems

We identified a total of 3,057 RMSs in the metagenome datasets with 432 assigned to type I RMS, 2,379 to type II RMS, and 246 to type III RMS. A normalization of these raw counts to copies per megabase (cpm) resulted in a similar distribution of RMS types I-III in the metagenomes. The sponge metagenomes showed higher abundances of all RMS types than seawater (2.48–5.08 cpm in sponges vs. 0.18 cpm in seawater). Type II was the most prevalent RMS type in the inspected metagenomes (*S. foetidus* = 4.03 cpm, *A. aerophoba* = 3.16 cpm, *P. ficiformis* = 2.06 cpm, seawater = 0.17).

**FIGURE 7:**
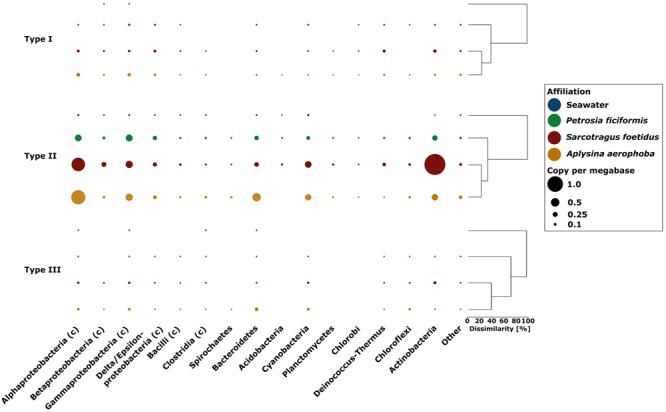
**Presence of types I–III restriction modification systems in the sponge and seawater metagenomes along with additional taxonomic assignments on the phylum and class (indicated through the c in brackets) level.** The size of each bubble indicates the gene copy number per megabase. Bray–Curtis dissimilarity for each RMS type is indicated by the dendrograms.

The majority of type I RMS genes were assigned to *Proteobacteria, Actinobacteria*, and *Deinococcus–Thermus* in the sponge metagenomes, while in seawater, type I RMS was assigned exclusively to the classes *Beta*- and *Gammaproteobacteria* (**Figure 7**). The majority of type II RMS in sponge metagenomes was assigned to *Proteobacteria (Alpha- and Gamma-)* and *Actinobacteria* as well as to a lesser extent, to *Bacteroidetes, Cyanobacteria*, and *Acidobacteria*. The *S. foetidus* metagenome contained an unusually high number of type II RMS affiliated to *Actinobacteria*. Type III RMS was the most underrepresented group. Type III RMS in sponge metagenomes was most represented by *Alpha-* and *Gamma-Proteobacteria* as well as *Bacteroidetes* and *Chloroflexi*, while type III in the seawater sample was only represented by the *Alpha*- and *Gammaproteobacteria, Bacteroidetes* and the *Clostridia*.

## Discussion

### General Features

We performed the taxonomic assignment of metagenomics reads by MG-RAST which has been previously attempted using microbial metatranscriptome data from a low microbial abundance sponge (Moitinho-Silva et al., 2014). While this approach offers the advantage of using the full metagenome dataset rather than a single gene marker (i.e., 16S rRNA gene), it may lose resolution for those phyla and candidate phyla where references genomes are not available. Our results confirm previous findings that sponges harbor a distinct microbiota which is different from that of the surrounding seawater. Principal component analysis of the relative abundance of reads revealed a clustering of the sponge samples (Supplementary Figure S3). The sponge metagenomes overlapped in their composition and they showed a higher proportion of *Actinobacteria* and *Deltaproteobacteria* than seawater based on assignment of complete metagenomic reads. In contrast, the seawater metagenome revealed higher abundances of *Alphaproteobacteria, Flavobacteria*, and *Cyanobacteria* compared to the sponge metagenomes. With increasing availability of sequence data and the completion of draft genomes by single cell genomics (Kamke et al., 2014) or binning approaches (Gao et al., 2014; Burgsdorf et al., 2015), the assignment of complete reads rather than single gene markers should become widely acceptable.

The sponge metagenomes displayed much higher GC contents (58–63%) than the seawater metagenome (41%) (**Figure 2A**). As has previously been recognized, the prokaryotic GC content can be highly variable between different environments (Foerstner et al., 2005; Reichenberger et al., 2015), ranging from 34% for Sargasso Sea surface water samples to 61% for terrestrial soils. The GC composition of the sponge metagenomes is much higher than most other metagenomes, only to be superseded by metagenomes from saline ponds and contaminated soils (Reichenberger et al., 2015). While an explanation for the variation in GC composition remains wanting, there is increasing evidence that both, the phylogenetic composition of the samples and the environment shape the GC composition of the resident microbiota. With respect to the sponge metagenomes, the GC contents are likely a result of bacterial community composition. *Actinobacteria*, which are known for their high GC content, are much more prevalent in the sponge metagenomes than in seawater. Accordingly, *S. foetidus* displayed the largest abundance of *Actinobacteria* (**Figure 1**) and the highest GC content (**Figure 2**). Nonetheless, this cannot be the only explanation, because in spite of variable abundances of *Actinobacteria* within the three sponges (**Figure 1**), the GC content is very narrow (**Figure 2A**). Therefore, we posit that the specific microenvironment within sponges has some yet to be characterized effect on the microbial GC composition of sponges.

The sponge metagenomes displayed larger calculated average genome sizes (3.0–5.1 Mb) than that calculated for seawater (1.35 Mb) (**Figure 2B**; **Table 2**). The estimates for sponge bacterial genomes are on the larger end of genome size estimates derived from diverse metagenomic data (Giovannoni et al., 2014). It should however be noted that the comparison of closely related *Synechococcus* genomes from sponge symbionts versus those from seawater did not reflect this pattern (Burgsdorf et al., 2015). Larger genomes of sponge-associated bacteria may be the evolutionary consequence of a more variable and nutrient-rich microenvironment within the sponge as opposed to the stable, nutrient poor seawater. Further the sponge-associated microbial consortia are constantly exposed to an ample source of free DNA resulting from the host’s digestion of food bacteria. Whether and to what extent the mechanisms of horizontal gene transfer occur in sponges and whether this would then results in larger symbiont genomes remains to be investigated in future studies. The high prevalence of transposases and other mobile genetic elements within sponge microbiomes (Fan et al., 2012) does suggest that horizontal gene transfer is rampant in the spongeholobiont.

The overall functional annotation on the level of COG categories was more similar within the sponge samples than compared to the seawater sample (**Figure 3**). The functional profile of *A. aerophoba* was more distant to the other sponge samples, which may have been influenced by a higher functional diversity as shown in the rarefaction curve (Supplementary Figure S2). Overall, only two COG categories were enriched in sponge metagenomes (defense mechanisms; cytoskeleton), while three COG categories were depleted in sponge metagenome over seawater (translation, ribosomal structure, biogenesis; cell motility; chromatin structure and dynamics). The category cytoskeleton was not pursued further owing to low gene abundance (<0.6 cpm). These results are somewhat different from previous data (Thomas et al., 2010), where the metagenome of the Australian sponge *C. concentrica* was enriched in two COG categories (secondary metabolites biosynthesis, transport and catabolism; replication, recombination, and repair) while being depleted in three other categories (translation, ribosomal structure and biogenesis; nucleotide transport and metabolism; energy production and conversion in comparison to seawater). The only shared feature between these analyses is the depletion of sponge metagenomes in the category: translation, ribosomal structure, biogenesis. The category defense mechanisms is discussed in detail below.

### Defense Systems

The overall enrichment of the category defense mechanisms in sponge metagenomes over seawater metagenome is in agreement with earlier results, where functions related to viral defense were found to be enriched in sponges (CRISPR-Cas system, RMS) over surrounding bacterioplankton (Thomas et al., 2010; Fan et al., 2012) or in selected bacterial reference genomes (Burgsdorf et al., 2015). The defense system DNA phosphorothioation was, even though not functionally complete, also more prevalent in the sponge metagenomes. Further, genes associated with phage growth limitation (Pgl) were enriched in the sponge metagenomes. The microbial consortia within sponges may thus not only defend themselves against viruses and phages, but may also be capable of suppressing their growth. Since the Pgl system is only poorly characterized, further studies are needed to fully understand its potential impact on microbial communities.

Clustered regularly interspaced short palindromic repeats arrays were identified through a protocol using three different tools to avoid false positive hits. The total set of arrays (and direct repeats) was 77, 47, 283, and 0 for the metagenomes of *P. ficiformis, S. foetidus, A. aerophoba* and seawater, respectively. While the CRISPR arrays in sponge metagenomes (0.21–0.62 cpm) are below the values described for completely sequenced genomes (0.72 cpm), they are still an order of magnitude above the values for seawater metagenomes, such as derived from the Sorcerer II Global Ocean Sampling expedition (0.042 cpm) (Sorokin et al., 2010). This value suggests a low number of CRISPRs in seawater and indeed, we found 0 hits in our seawater metagenome. The variation in the number of observed CRISPR arrays between the sponge metagenomic datasets may be due the fragmentation of generated contigs and is based on the used sequencing technology and the assembly algorithms (Gogleva et al., 2014).

The overall taxonomic assignment (>68.83%) was comparable between the datasets with the largest fraction of CRISPR arrays affiliated to *Proteobacteria* followed by *Actinobacteria, Chloroflexi* and *Firmicutes* (**Table 3**). Similar results were observed for the origin of the spacer sequences (**Figure 6**). While this finding supports the presence of similar microbiomes within the different sponge species, only a small overlap of repeat sequences was identified. As ∼30% of direct repeats could not be assigned to a superclass or known repeats, they may represent novel direct repeats. The fact that we did not detect any shared spacers suggests that the acquisition of protospacers may vary between bacterial individuals (Gogleva et al., 2014). The sponge-associated bacteria may either be exposed to different types of viruses, phages or plasmids (Burgsdorf et al., 2015) or to distinct viral variations (Fan et al., 2012). With respect to the targets of the spacer sequences, their number decreased from the group “unknown” to plasmids, phages and viruses, and they were uniformly distributed among all identified phyla (**Figure 6**). The large fraction of hits to unknown and unique spacer sequences suggests that a large number of novel and diverse CRISPR targets and spacers can be expected in marine sponge metagenomes. The small overlap between spacers and direct repeats of the CRISPR-Cas systems likely reflects variations within each sponge metagenome as well as the specific acquisition of spacers from selected bacteria.

We found only ∼50% of all CRISPR arrays adjacent to *cas* genes, which is likely an effect of the fragmentation of the assemblies. The *cas* genes were used to classify CRISPR systems into types and subtypes according to Makarova et al. (2011). Overall and as was expected, *cas1* (an universal marker of all CRISPR-*cas* systems) and *cas2* were most prevalent. CRISPR-Cas type I, described via *cas3*, was the most prevalent in all three sponge metagenomes followed by types II and III, identified by *cas9* and *cas10*. The latter two types were only found in very low abundances, suggesting type I to be the most important CRISPR type in the sponge microbiome. Interestingly, type I was also most prevalent in other environments such as the human gut (Gogleva et al., 2014) or groundwater (Burstein et al., 2016). The most abundant subtypes I–E showed a strong link to *Actinobacteria* (Makarova et al., 2015). In ecological terms, the high prevalence of CRISPR-Cas systems in sponge microbiomes may be necessary to defend the sponge-associated bacteria against viral particles that are drawn into the sponge holobiont by filtration. It has previously been estimated that the sponge-associated bacteria may be exposed to as many as 1000 viral particles per day (Thomas et al., 2010), thus an efficient defense against viral onslaught could be essential.

Restriction modification system have previously been shown to be more abundant in metagenomes from Australian sponges than in seawater (Fan et al., 2012). We here confirm these results for the Mediterranean sponges (2.48–5.08 cpm vs. 0.18 for RMS in seawater metagenome). The difference might be explained by the observation that larger genomes tend to have more RMS than smaller genomes (Makarova et al., 2013), which is indeed the case for the sponge metagenomes over the seawater metagenomes (**Figure 2B**). Among the different types of RMS, type II was most abundant in the metagenomes (**Figure 7**) which is consistent with previous findings for bacterial isolates (Oliveira et al., 2014). Similar to CRISPR, the RMS are mostly affiliated with *Alphaproteobacteria, Gammaproteobacteria, Betaproteobacteria*, and *Actinobacteria*. Both CRISPR and RMS thus appear as the first line of defense against foreign DNA, in particular against attack by viruses or phages.

## Conclusion

A comparison of microbial metagenomes from different Mediterranean sponge species versus seawater revealed bacterial defense systems as the consistently enriched feature in sponge metagenomes. These defenses include CRISPRs, RMSs, phage growth inhibition and DNA phosphorothioation as the main mechanisms to combat foreign DNA from viruses, phages or other sources. The expanded genomic repertoire for bacterial defenses is likely the result of an evolutionarily long-standing adaptation where the resident sponge microbiota is exposed to free DNA resulting from the immense filtration activities of the animal host. In support of this, higher GC contents and larger calculated genome sizes were identified in sponge metagenomes over seawater. Collectively, our results indicate that the genomes of sponge microorganisms are/have been subject to horizontal gene transfer and that defense against foreign DNA is one prerequisite for an existence within sponges.

## Author Contributions

HH designed the study, carried out metagenomics analysis, prepared figures and drafted the manuscript. BS collected the *A. aerophoba* sample and carried out its analysis. MJ participated in the analysis, preparation of figures and study design. KB sampled *P. ficiformis* and seawater and helped in the study design. LM-S sampled *S. foetidus* and participated in the study design. FF carried out analysis and wrote scripts. UA participated in the study coordination and drafting of the manuscript. UH coordinated the study and drafted/finalized the manuscript. All authors read and approved the final manuscript.

## Conflict of Interest Statement

The authors declare that the research was conducted in the absence of any commercial or financial relationships that could be construed as a potential conflict of interest.

## Footnotes

1 http://www.bioinformatics.babraham.ac.uk/projects/fastqc/

2 https://sourceforge.net/projects/bbmap/

3 https://gold.jgi.doe.gov/biosamples?Study.GOLD%20Study%20ID=Gs0099546

## Abbreviations

CRISPR: clustered regularly interspaced short palindromic repeats
MTase: methyltransferase
nt: NCBI nucleotide database
nr: NCBI non-redundant protein database
REase: restriction endonuclease
RMS: restriction modification system
