# Supplementary material for: An Enrichment of CRISPR and Other Defense-Related Features in Marine Sponge-Associated Microbial Metagenomes
Source: Front Microbiol. 2016 Nov 8;7:1751. doi: 10.3389/fmicb.2016.01751 (PMC5099237; doi:10.3389/fmicb.2016.01751)
Supplement: Supplementary file 1 [file Data_Sheet_1.DOCX]

Supplementary Material

An enrichment of CRISPR and other defense-related features in marine sponge-associated microbial metagenomes

Hannes Horn^1,2^, Beate M. Slaby^1^, Martin T. Jahn^1^, Kristina Bayer^1^, Lucas Moitinho-Silva^3^, Frank Förster^4^, Usama R. Abdelmohsen^2^, Ute Hentschel^1,5,*^

^1^ GEOMAR Helmholtz Centre for Ocean Research, RD3 Marine Microbiology, D-24105 Kiel, Germany

^2^ University of Würzburg, Department of Botany II, Julius-von-Sachs Institute for Biological Sciences, D-97082 Würzburg, Germany

^3^ Centre for Marine Bio-Innovation, University of New South Wales , Sydney, New South Wales , Australia

^4^ University of Würzburg, Department of Bioinformatics, D-97082 Würzburg, Germany

^5^ Christian-Albrechts-University of Kiel, D-24118 Kiel, Germany

***Correspondence:**Ute Hentschel
[uhentschel@geomar.de](mailto:uhentschel@geomar.de)

# Supplementary Figures

**
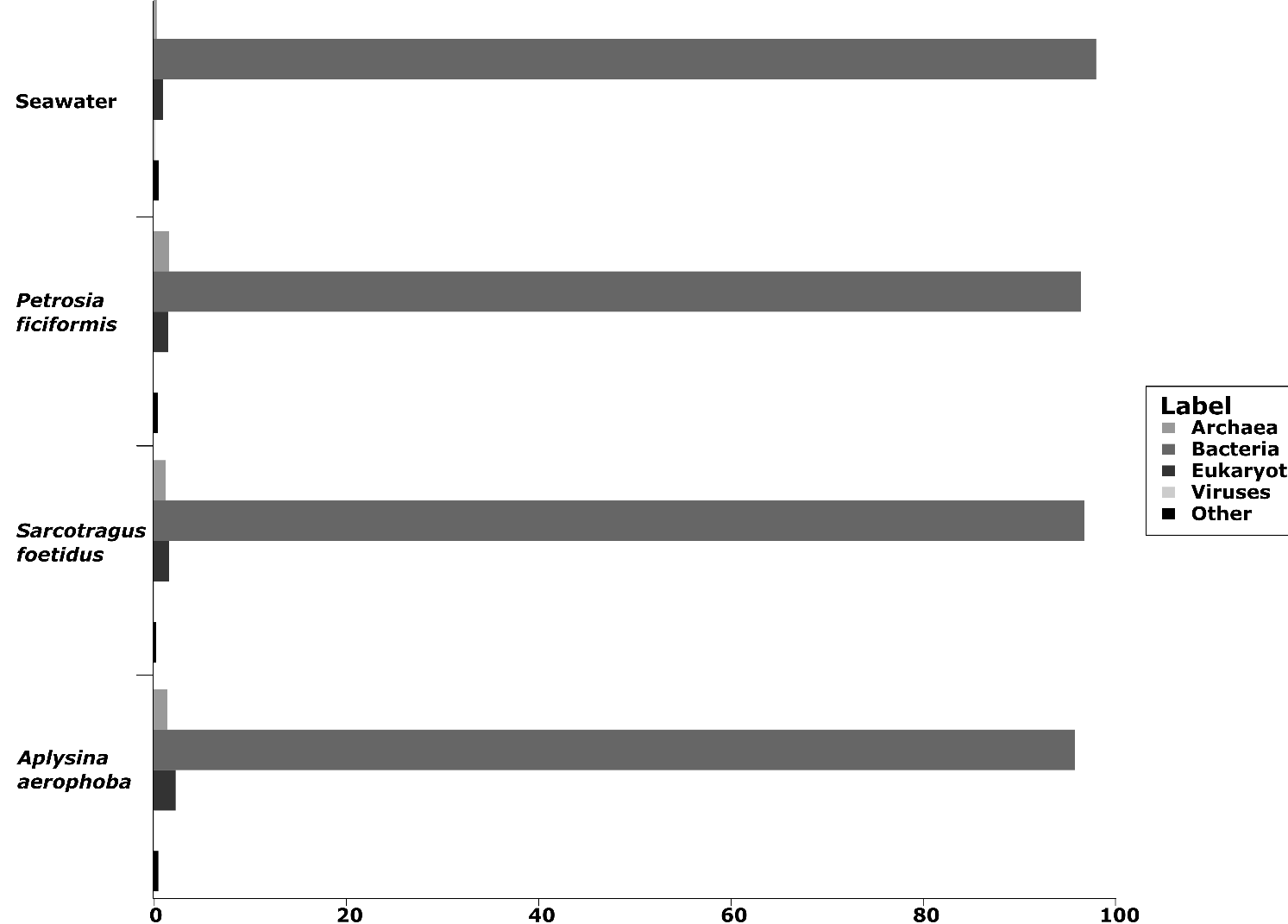
**

**Figure S1** – Barplot showing the relative genomic diversity on the phylum level. The relative abundance is scaled by the x-axis. Taxonomic assignments are based on MG-Rast assignments. The group ‘other’ comprises unclassified sequences.


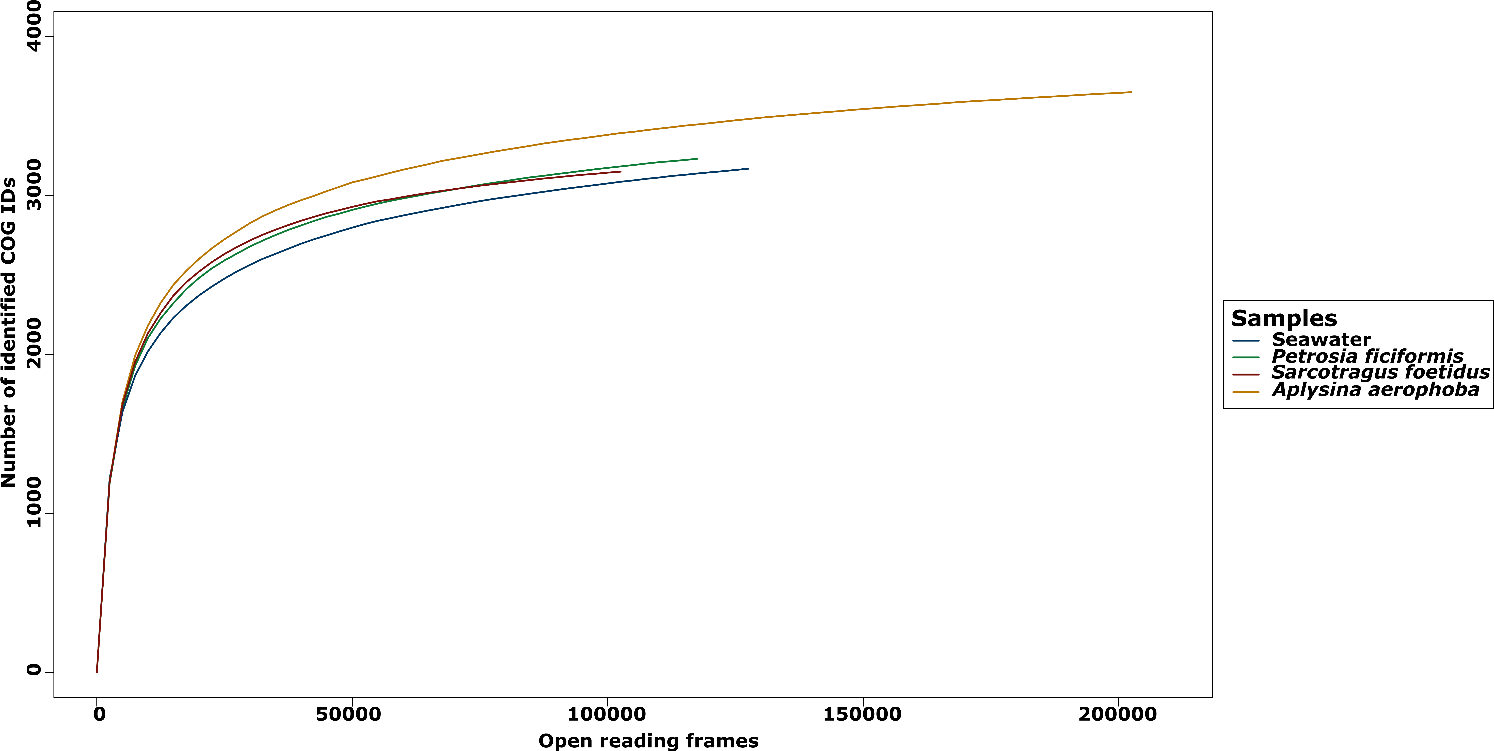


**Figure S2** – Rarefaction curve showing the diversity of COG functional gene categories identified in the four metagenomes. Each curve represents an individual metagenome. The number of sampled open reading frames is indicated on the x-axis.


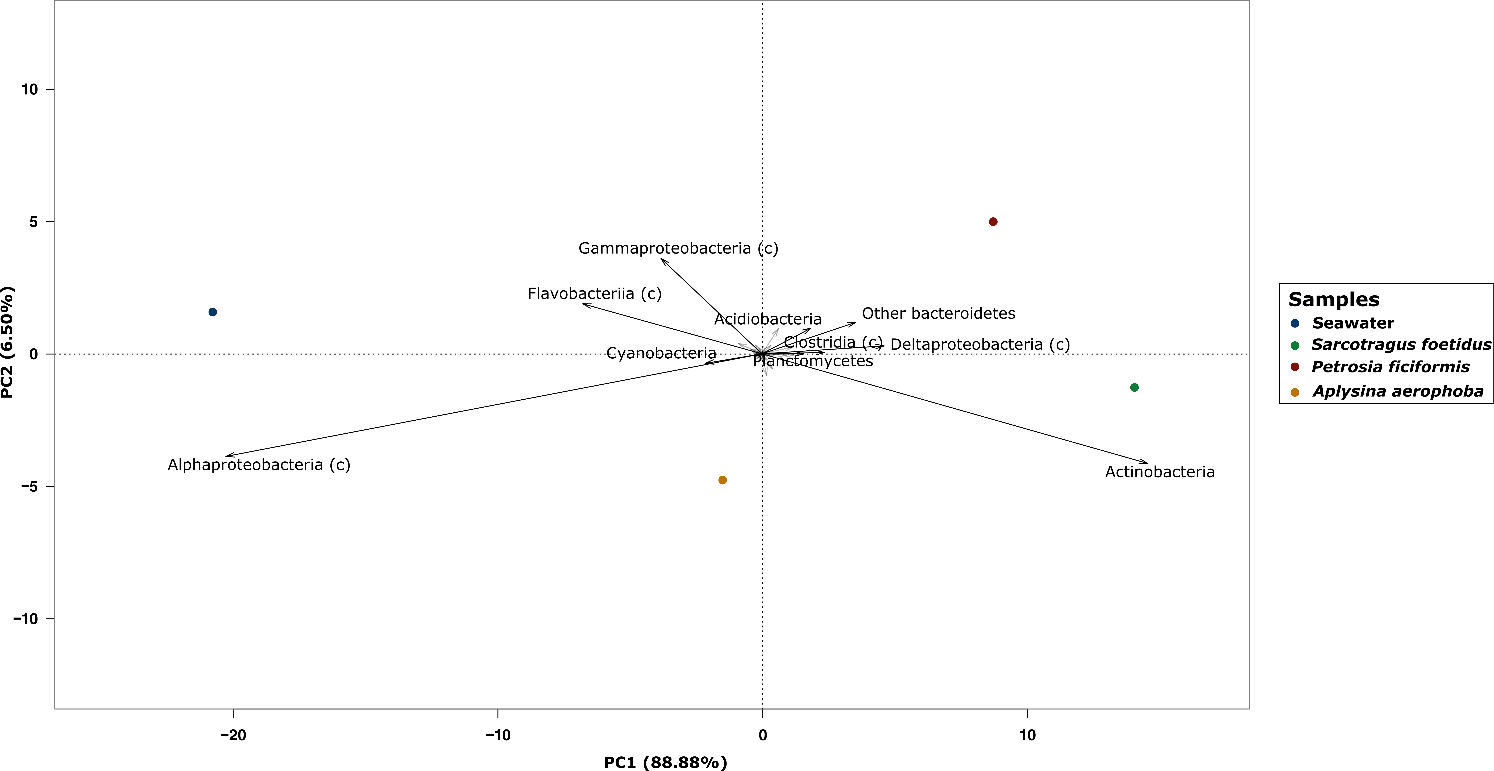


**Figure S3** – Principal component analysis (PCA) of the relative abundance of taxonomic assignments of the phylum and class level of the four analyzed metagenomes. Shown are the first two axes (PCA1, PCA2) and the ten highest influencing variables.
